# *KRAS*突变型非小细胞肺癌的免疫治疗进展

**DOI:** 10.3779/j.issn.1009-3419.2025.101.08

**Published:** 2025-05-20

**Authors:** Xinyue YANG, Zhiwei TANG, Li MA, Ran CHEN

**Affiliations:** ^1^441000 襄阳，武汉科技大学医学院襄阳市第一人民医院研究生联合培养基地肿瘤科; ^1^Department of Oncology, Postgraduate Union Training Base of Xiangyang No.1 People's Hospital, School of Medicine, Wuhan University of Science and Technology, Xiangyang 441000, China; ^2^441000 襄阳，湖北医药学院附属襄阳市第一人民医院肿瘤科; ^2^Department of Oncology, Xiangyang No.1 People's Hospital, Hubei University of Medicine, Xiangyang 441000, China; ^3^101149 北京，首都医科大学附属北京胸科医院北京市结核病胸部肿瘤研究所肿瘤内科; ^3^Department of Medical Oncology, Beijing Chest Hospital, Capital Medical University, Beijing Tuberculosis and Thoracic Tumor Research Institute, Beijing 101149, China

**Keywords:** 肺肿瘤, *KRAS*突变, 免疫治疗, 免疫检查点抑制剂, Lung neoplasms, *KRAS* mutation, Immunotherapy, Immune checkpoint inhibitors

## Abstract

在流行病学统计中肺恶性肿瘤的发病率与致死率均位居前列。非小细胞肺癌（non-small cell lung cancer, NSCLC）是肺癌的重要组成部分，成为临床研究与治疗的重点关注对象。在NSCLC基因组学特征中，Kirsten大鼠肉瘤病毒基因同源物（Kirsten rat sarcoma viral oncogene homolog, *KRAS*）突变属于主要的肿瘤驱动因素之一，约占所有NSCLC病例的25%。该突变的存在与患者的治疗反应及预后密切相关，因此针对*KRAS*突变型NSCLC的治疗策略是肿瘤研究领域的重要课题。当前时代背景下，免疫调节治疗凭借其独特的作用机制与显著的临床疗效在肿瘤学领域快速普及并迅速发展，针对NSCLC *KRAS*驱动基因突变亚型的治疗策略逐渐成为研究热点。免疫检查点抑制剂（immune checkpoint inhibitors, ICIs）的诞生，为这类癌症患者开辟了全新的治疗路径，临床研究显示其在改善生存期方面具有显著效果。然而，目前在免疫治疗的应用中仍面临诸多挑战，如肿瘤微环境的复杂性、患者个体差异及耐药机制等。本文综述了*KRAS*突变型NSCLC的免疫治疗进展，重点分析了免疫治疗的具体应用、联合疗法的探索及其相关临床试验结果，同时探讨了*KRAS*突变型NSCLC治疗未来可能的发展方向，为临床治疗实践提供参考。

## Abstract (en)

In epidemiological statistics, the incidence rate and mortality rate of malignant lung tumors rank among the top. Non-small cell lung cancer (NSCLC) constitutes an important part of lung cancer and has become a key focus of clinical research and treatment. Among the genomic characteristics of NSCLC, the Kirsten rat sarcoma viral oncogene homolog (*KRAS*) mutation is one of the main tumor drivers, accounting for approximately 25% of all NSCLC cases. The existence of this mutation is closely related to the treatment response and prognosis of patients. Therefore, the treatment strategy for *KRAS*-mutated NSCLC is an important topic in the field of tumor research. In the current era, immunomodulatory therapy has rapidly gained popularity and developed rapidly in oncology due to its unique mechanism of action and remarkable clinical efficacy. The treatment strategies targeting the *KRAS*-mutated of NSCLC have gradually become a research hotspot. The advent of immune checkpoint inhibitors (ICIs) has opened up a new therapeutic avenue for patients with such cancers, and clinical studies have shown significant effects in improving survival rates. Nevertheless, there are still many challenges in the application of immunotherapy, such as the complexity of the tumor microenvironment, individual differences among patients, and drug resistance mechanisms. This article reviews the progress of immunotherapy for *KRAS*-mutated NSCLC, focusing on the specific application of immunotherapy, the exploration of combination therapies, and the results of related clinical trials. At the same time, it discusses the possible future development directions of *KRAS*-mutated NSCLC treatment, providing a reference for clinical treatment practice.

肺癌在恶性肿瘤中具有极高的致死率，位居癌症相关死亡首位，同时其新发病例数在全球范围内位列第二^[1]^，其侵袭性肿瘤特征不仅直接构成生命威胁，治疗产生的并发症及经济状况均与患者的预后密切相关。肺癌在组织学上可划分为小细胞肺癌与非小细胞肺癌（non-small cell lung cancer, NSCLC），NSCLC在肺癌病例中占比显著^[1]^。在NSCLC的组织学亚类中，肺腺癌（lung adenocarcinoma, LUAD）的发病率显著高于其他病理类型，比例接近半数；其次是肺鳞癌（lung squamous cell carcinoma, LUSC），占比约三成^[2]^。

Kirsten大鼠肉瘤病毒基因同源物（Kirsten rat sarcoma viral oncogene homolog, *KRAS*）基因隶属于大鼠肉瘤病毒性肿瘤基因家族（rat sarcoma viral oncogene homolog, *RAS*），该驱动基因在各类癌种中的突变谱占比最为突出^[3]^。*KRAS*是NSCLC中常见的致癌驱动基因^[4]^，*KRAS*突变在NSCLC中具有显著的流行病学特点，其发生率因肺癌的病理类型、种族、吸烟史和性别而显著不同^[5]^。NSCLC中*KRAS*基因特异性改变有很大概率影响肿瘤生物学特性和预后^[6]^。有研究^[7]^表明，在剔除表皮生长因子受体突变这一情况后，*KRAS*突变型NSCLC有更差的无进展生存期（progression-free survival, PFS）和总生存期（overall survival, OS）。*KRAS*突变的分布在不同的肿瘤类型和患者群体中也存在差异，这可能与遗传背景、环境因素以及肿瘤微环境（tumor microenvironment, TME）的特征等因素有关^[8]^。因此，了解*KRAS*突变在NSCLC中的流行病学特征，对于指导临床精准治疗策略的制定及优化患者临床结局具有至关重要的临床指导价值。

免疫治疗领域的突破性进展为NSCLC患者的临床治疗开辟了崭新维度，靶向免疫检查点的创新疗法在多项临床治疗中展现出令人瞩目的治疗潜力，逐渐成为晚期NSCLC患者治疗的关键手段。与NSCLC的传统放疗、化疗等手段相比，免疫检查点抑制剂（immune checkpoint inhibitors, ICIs）加入临床治疗后，显著提升了部分患者的预后效果^[9]^。在一项二线治疗研究（CheckMate057）中，与多西他赛（Docetaxel）化疗相比，使用纳武利尤单抗（Nivolumab）的*KRAS*突变型NSCLC患者的中位OS有所改善^[10]^。这一结果不仅证实了免疫治疗在*KRAS*突变型NSCLC患者的二线治疗中的优势，也为临床医生提供了更有效的治疗选择。然而，尽管部分*KRAS*变异的肺癌患者在免疫治疗中表现出积极的临床响应，但仍存在许多挑战，如肿瘤的免疫逃逸机制及治疗耐药性等问题^[11]^。这些挑战促使我们进一步探索如何更有效地结合免疫治疗与其他治疗手段，如免疫治疗与传统的化疗、放疗相结合，利用化疗和放疗对肿瘤细胞的直接杀伤作用，同时借助免疫治疗激活免疫系统，增强机体对肿瘤细胞的长期免疫监视；免疫治疗与靶向治疗的联合应用也值得深入研究，针对*KRAS*突变等特定的分子靶点，开发更精准的联合治疗方案，以提高*KRAS*突变型NSCLC患者的治疗效果，对免疫治疗策略进行不断优化和创新，为*KRAS*突变型NSCLC患者带来更好的生存预后和生活质量。

## 1 *KRAS*基因概述

### 1.1 *KRAS*基因及其突变类型

*KRAS*属于人类出现突变频率较高的癌基因，尤其在NSCLC、结直肠癌和胰腺导管腺癌等多种肿瘤中频繁出现。*KRAS*基因突变在人类恶性肿瘤中呈现高频发生态势，约占LUAD的1/5^[12,13]^。不同癌症类型中*KRAS*突变的分布也有差异，在NSCLC患者中*KRAS*突变的发生率约为25%，其中大多数突变发生在12和13号密码子，突变类型有G12C、G12S、G13C等^[14]^，G12C占所有*KRAS*突变的40%，G12V和G12D突变则分别占20%和15%^[15,16]^，而在胰腺导管腺癌中最普遍的是G12D和G12V^[17]^。*KRAS*突变不仅影响肿瘤的发生和发展，还与患者的预后密切相关^[8]^。有研究^[18,19]^表明，在*KRAS*突变的晚期NSCLC患者中G12C突变亚型与*KRAS*其他突变亚型相比预后差，但也有研究^[20]^表明，在*KRAS*突变的亚型中，G12C组患者较其他突变亚型组，OS和PFS未见显著差异。因此临床研究的不同发现表明，对于不同*KRAS*突变亚型NSCLC患者的预后情况尚不明确，还需要进一步研究与探讨。

### 1.2 *KRAS*突变对肿瘤生长及转移的影响

*KRAS*相较于其他小GTP酶家族成员，在细胞复制、分化及生存的调控网络中产生不可替代的作用^[3]^。在结直肠癌中，*KRAS*突变与肿瘤的转移性进展密切相关，突变型展现出更为显著的迁移能力，侵袭性表征也更为突出^[21]^。在一项研究^[22]^中，*KRAS* G12C突变的肿瘤模型显示出较高的侵袭性与迁移能力，表明这种突变可能通过上调多种转移相关基因来促进肿瘤的转移。*KRAS*蛋白被激活后导致RAF-MEK-ERK通路、PI3K-AKT-mTOR通路和RAS相关蛋白（Ral-GEF）通路这3个关键通路的下游信号转导^[23]^。*KRAS*激活导致下游效应子的募集和激活，这些效应子可以进一步激活下游蛋白质，部分可作为转录因子并进入细胞核，导致调节细胞增殖、存活和血管生成的基因表达，促进细胞的增殖和生存，从而推动肿瘤的进展^[24,25]^。*KRAS*突变虽然通过上述信号通路发生作用，但不同亚型的突变对通道的依赖性不同，*KRAS* G12C更依赖于通过RAF-MEK-ERK信号通路传导来增加致癌潜力并促进肿瘤生长，而*KRAS* G12D更依赖于PI3K-AKT-mTOR信号传导^[26]^。*KRAS*突变表现出不同的生物学特性，因此，明确*KRAS*突变的类型及其对肿瘤生物学的影响，对临床精准治疗策略的制定具有不可或缺的指导价值。

### 1.3 *KRAS*突变与TME的相互作用

*KRAS*在NSCLC中发挥着重要作用，它不仅影响肿瘤细胞的增殖和存活，还能通过多种机制改变TME的组成和功能。*KRAS*突变在TME中诱导促炎状态的形成，*KRAS*使白细胞介素6和白细胞介素8的分泌增加，激活ERK89通路等相关通路，促使TME中的炎症的发生^[27]^。*KRAS*相关通路与各种自身免疫性疾病的发展也有关，当*KRAS*信号通路过度激活时可能会导致类风湿性关节炎、系统性红斑狼疮等疾病的发生^[28]^。*KRAS*突变还与免疫抑制微环境的形成有关，影响免疫细胞的浸润和活性，导致抑制性免疫细胞如调节性T细胞和髓源性抑制细胞等在TME中的积累，从而削弱了抗肿瘤免疫反应，导致肿瘤进展^[29]^。Pinto等^[30]^研究发现，在*KRAS*突变的LUAD患者中B细胞浸润减少，这可能是其导致免疫抑制的原因。通过对肿瘤免疫浸润数据库（Tumor Immune Estimation Resource, TIMER, URL: http://timer.cistrome.org/）分析发现，NSCLC TME中*KRAS*的表达水平与CD8^+^ T细胞、巨噬细胞、中性粒细胞、树突状细胞等关键免疫细胞的富集程度存在显著正向关联，也间接说明*KRAS*突变伴随着关键免疫细胞高浸润水平，预示着存在较好的免疫治疗疗效。这些发现为开发针对*KRAS*突变的免疫治疗策略提供了新的思路，尤其是在结合*KRAS*抑制剂和ICIs的联合治疗方面。

## 2 ICIs在*KRAS*突变型NSCLC中的应用

### 2.1 主要ICIs及其临床应用

ICIs是一类通过阻断免疫检查点分子来激活机体免疫系统，从而对抗肿瘤的药物。目前，已批准多种ICIs用于治疗NSCLC，这些药物主要作用于CD279/程序性细胞死亡受体1（programmed cell death protein 1, PD-1）和CD274/程序性细胞死亡配体1（programmed cell death-ligand 1, PD-L1），代表性药物包括阿替利珠单抗（Atezolizumab）、纳武利尤单抗和帕博利珠单抗（Pembrolizumab）；以及靶向细胞毒性T淋巴细胞抗原4通路的伊匹木单抗（Ipilimumab）。T细胞被激活后在细胞膜表面呈现PD-L1的高表达状态，与其相结合抑制T细胞增殖，减少细胞因子产生，降低细胞溶解活性，从而影响T细胞发挥作用，ICIs通过抑制PD-L1结合，重新激活肿瘤特异性T细胞免疫^[31]^。近年来，ICIs已被应用到多种肿瘤的治疗，为广大患者带来美好前景。

### 2.2 *KRAS*突变与PD-L1表达的相关性

近年来，抗PD-1和抗PD-L1的免疫治疗为NSCLC患者带来了重大希望，PD-L1是上述治疗的优势人群选择的最常用标志物。而有研究^[32]^表明，*KRAS*或TP53突变型NSCLC中的PD-L1蛋白表达水平高于非突变型肿瘤，这表明*KRAS*可能通过PD-L1参与免疫系统抑制。当*KRAS*发生突变时，持续处于激活状态，从而激活RAF-MEK-ERK信号通路，通过激活ERK磷酸化信号传导途径，促进PD-L1的转录，PD-1/PD-L1轴诱导CD3^+ ^T细胞凋亡，细胞毒性T细胞被PD-L1灭活，导致TME的免疫监视受限，进而影响TME中的免疫逃逸机制^[33,34]^。基于肿瘤免疫浸润数据库TIMER的分析显示，NSCLC患者的*KRAS*和PD-L1的表达水平呈正相关。肿瘤突变负荷（tumor mutational burden, TMB）是抗PD-1治疗效果评估的最新标志物，反映了免疫治疗的临床效果，*KRAS*突变患者的TMB通常较高^[35]^，这表明*KRAS*突变的患者对ICIs反应可能较好。在*KRAS*突变型NSCLC中高PD-L1表达患者有望通过免疫治疗获得更好的预后。

### 2.3 *KRAS*突变与ICIs疗效的关系

目前，ICIs已经在多种肿瘤治疗中表现出明显效果。*KRAS*突变在NSCLC患者中具有重要的预后意义，此突变可能与ICIs的疗效密切相关。在一项单中心、回顾性队列研究^[36]^中，*KRAS*变异型在进行ICIs干预时，*KRAS* G12C突变的患者通常表现出更好的临床反应，与其他*KRAS*突变患者之间的OS存在显著差异（分别为7.7和6.0个月；P=0.018）。而*KRAS* G12D变异阳性的NSCLC治疗人群通过下调PD-L1水平和CD8^+^肿瘤浸润淋巴细胞（CD8^+^ tumor infiltrating lymphocytes, CD8^+^ TILs）在NSCLC中的浸润，驱动免疫抑制和针对CD279/CD274靶向干预的原发性耐药，其PFS明显低于具有其他突变亚型的患者^[37]^。*KRAS*突变促进对ICIs的耐药机制可能涉及免疫抑制细胞因子的产生，例如血管内皮生长因子，以及对T细胞募集的抑制作用^[38]^。在一项回顾性研究^[39]^中，*KRAS* G12C突变患者在接受二线免疫治疗的PFS显著高于其他野生型患者，*KRAS* G12C突变患者的中位PFS为23个月，而非突变患者的中位PFS仅为5个月（HR=3.28, 95%CI: 0.86-12.5, P=0.03）。而在另一个回顾性研究^[40]^中，在应用ICIs时，*KRAS* G12C突变的NSCLC患者OS和PFS与*KRAS*野生型患者相比无明显差异。这可能与*KRAS*突变的类型以及ICIs的类型有关，需要对其可能涉及的其他变量进行进一步探索。免疫药物在临床试验中显示出对*KRAS*突变型患者优越的抗肿瘤活性，尤其是在PD-L1表达高的患者中^[41]^。在PD-L1高表达的患者（≥50%）中突变率显著高于低表达患者（1%-49%），这一现象可能与*KRAS*突变诱导PD-L1表达有关^[42]^。许多相关免疫治疗的进展正在被报道，其中有关于*KRAS*驱动基因阳性的NSCLC使用ICIs治疗的相关临床研究^[40,43-48]^数据汇总见表1。这些发现强调了在这些患者中，不同突变亚型对于ICIs疗效有显著差异，表明为此类群体制定个体化治疗策略的重要性。

**表 1:** 免疫检查点抑制剂在*KRAS*突变型NSCLC中的疗效

Reference	Treatment method/ Study type	*KRAS* subtype	Trail design (endpoints, number of patient)	Outcomes (ORR, DCR, mPFS, mOS)
Zhao *et al*.^[43]^	First-line ICIs/ Retrospective study	G12C, G12A, G12D, G12V, other	ORR, mOS *n*=25 (Responders=13; Non-responders=12)	ORR: 52% mOS (Responders: 26.3 mon; Non-responders: 15.6 mon)
Wu *et al*.^[44]^	First-line ICIs/ Retrospective study	G12C, Non-G12C	mOS *n*=23	mOS: 35.6 mon
Uehara *et al*.^[45]^	First-line ICIs/ Retrospective study	G12C, G12D, G12V, G12S, G13C, other	mPFS, mOS *n*=21	mPFS: 16.2 mon mOS: 31.3 mon
Kartolo *et al*.^[46]^	First-line ICIs/ Retrospective study	G12C, G12A, G12D, G12V, G12S, other	ORR, mPFS, mOS *n*=30	ORR: 37% mPFS: 6.0 mon mOS: 12.9 mon
Passiglia *et al*.^[47]^	First-line ICIs/ Retrospective study	ND	ORR, DCR, mPFS, mOS *n*=206	ORR: 20% DCR: 47% mPFS: 4.0 mon mOS: 11.9 mon
Jiang *et al*.^[48]^	First-line ICIs/ Retrospective study	G12C, G12D, G12S, other	mPFS, mOS *n*=110	mPFS: 7.6 mon mOS: 18.5 mon
Jeanson *et al*.^[40]^	First-line ICIs/ Retrospective study	G12A, G12C, G12D, G12V, G13C	ORR, DCR, PFS, OS *n*=162	ORR: 18.7% DCR: 48.4% mPFS: 3.09 mon mOS: 14.29 mon

*KRAS*: Kirsten rat sarcoma viral oncogene homolog; NSCLC: non-small cell lung cancer; ICIs: immune checkpoint inhibitors; ORR: objective response rate; mPFS: median progression-free survival; mOS: median overall survival; DCR: duration of response; ND: not described.

## 3 联合疗法的研究进展

### 3.1 免疫治疗与化疗的联合应用

近年来，免疫治疗与化疗的联合应用在NSCLC等多种癌症的治疗中显示出了良好的前景。Zhou等^[49]^通过回溯性临床数据揭示了转移性NSCLC患者接受初始免疫治疗后进展的特征，并探讨了放疗协同作用的临床价值，该研究发现，在首选免疫治疗方案疗效不佳的群体中，序贯性胸廓放射干预可产生协同效应。在免疫治疗和化疗联合治疗之前，针对NSCLC伴*KRAS*基因变异的病例，临床治疗方案通常以化疗为主导，其次是ICIs的开发和使用^[50,51]^。PD-L1或*KRAS*沉默后不仅使LUAD细胞的增殖减少，凋亡增加，还提高了紫杉醇的细胞毒性，使细胞对化疗的敏感性增加^[52]^，因此*KRAS*突变的患者同时靶向PD-L1和*KRAS*联合化疗的治疗方案值得我们关注。Merle等^[53]^研究也表明，ICIs（如PD-1和PD-L1抑制剂）与化疗药物的联合使用能够增强抗肿瘤免疫反应，提高NSCLC患者的生存率。一项对6项研究的荟萃分析^[54]^显示，与单纯使用细胞毒性药物，免疫治疗与其联合显著延长了*KRAS*突变型NSCLC受治者的OS（HR=0.59, 95%CI: 0.49-0.72; *P*<0.00001）和PFS（HR=0.58, 95%CI: 0.43-0.78; P=0.0003），且OS也显著增加（P=0.001）。在KEYNOTE-189试验^[55]^中，对研究结果深入剖析发现，出现任何*KRAS*突变的NSCLC患者在接受帕博利珠单抗联合化疗时比单独接受化疗的患者获得了更好的结局。而又有研究^[37]^结果表明ICIs联合化疗对*KRAS* G12D突变的NSCLC患者可能更有效。而不同类型的*KRAS*突变疗效也大不相同，针对不同的TME和基因突变背景，个体化的联合治疗方案也在不断探索中，以期最大限度地提高*KRAS*突变的NSCLC患者治疗效果并减少副作用。

### 3.2 免疫治疗与靶向治疗的联合策略

近年来，随着医学的发展，免疫治疗与靶向治疗的联合策略成为癌症治疗的重要研究方向。两者的作用机制不同，靶向治疗能够针对特定的肿瘤驱动基因来发挥抗肿瘤作用，而免疫治疗则通过调节机体免疫系统来对抗肿瘤。*KRAS*突变与不良预后相关，近年来针对*KRAS* G12C突变的靶向治疗取得了显著进展，例如索托拉西布和阿达格拉西布（Adagrasib）等药物被美国相关监管机构批准实施在临床应用中^[56]^。在一项II期研究^[57]^中，共纳入174例*KRAS* G12C突变局部晚期或转移性NSCLC患者，针对这些患者，给予索托拉西布960 mg *qd*进行治疗，直至患者病情出现进展，研究结果显示这些NSCLC患者的客观缓解率达到了40.7%，疾病控制率为83.7%，中位缓解持续时间为12.3个月，中位PFS为6.3个月，中位OS为12.5个月，1年OS率为50.8%。尽管靶向治疗在临床应用中具有初步疗效，但绝大多数患者都会产生治疗耐药性；而单用ICIs的疗效存在明显局限性，因此免疫治疗和靶向治疗联合的治疗方案逐渐成为*KRAS*突变的NSCLC患者的研究重点。在相关临床研究中，*KRAS* G12C突变的NSCLC患者在接受靶向药物（如索托拉西布）治疗的同时，结合ICIs能够显著提升疗效，延缓肿瘤进展^[58,59]^。在存在多重突变的肿瘤中，其免疫治疗和靶向治疗过程中有时会出现耐药的现象，因此免疫治疗和靶向治疗联合的治疗方案值得我们继续探索。

### 3.3 联合治疗的现状和未来发展

免疫治疗与化疗的联合使用使得*KRAS* G12C突变NSCLC患者中位OS延长，且PFS也有显著提升，联合治疗的反应率高达65%^[60]^。目前对于*KRAS*突变的NSCLC患者相关临床试验和回顾性研究在不断地进行，我们对“*KRAS* mutation、Immunotherapy、NSCLC”等关键词在PubMed数据库中进行检索，对获得的相关*KRAS*突变NSCLC免疫联合治疗的文献进行汇总见表2^[20,55,61-63]^。在部分临床试验中，联合治疗组的客观缓解率较单药治疗有了显著提升，并且疾病控制率也维持在较高水平。但联合治疗并非尽善尽美，在治疗过程中，部分患者会出现免疫相关不良反应，如免疫性肺炎、内分泌紊乱等。这些治疗相关毒性反应不仅可能降低患者的用药配合度，在极端情况下其潜在风险可直接导致生命危险。因此，未来在实行联合治疗方案时，需要根据免疫治疗、化疗、靶向治疗等特点及不良反应来制定合适的联合治疗方案，最大程度地发挥联合治疗的优势，同时降低不良反应的发生风险。

**表 2:** *KRAS*突变型NSCLC免疫联合治疗的疗效

Reference	Treatment method/ Study type	*KRAS* subtype	Trail design (endpoints, number of patient)	Outcomes (ORR, DCR, mPFS, mOS)
West HJ *et al*.^[61]^	Immunotherapy combined treatment/ Prospective study phase III	ND	mPFS, mOS *n*=74	mPFS: 4.8 mon mOS: 11.7 mon
Sun *et al*.^[62]^	Immunotherapy combined treatment/ Retrospective study	ND	mOS *n*=201	mOS: 20.0 mon
Li *et al*.^[63]^	Immunotherapy combined treatment/ Retrospective study	G12A, G12V, G12C, G12D, Other	mPFS *n*=23	mPFS: 12.8 mon
Gadgeel *et al*.^[55]^	Immunotherapy combined treatment/ Retrospective study	G12C	ORR, mPFS, mOS *n*=26	ORR: 50% mPFS: 11.0 mon mOS: 18.0 mon
Gu *et al*.^[20]^	Immunotherapy combined treatment/ Retrospective study	ND	ORR, DCR, mPFS *n*=33	ORR: 27.2% DCR: 93.9% mPFS: 7.4 mon

## 4 免疫治疗在*KRAS*突变型NSCLC患者的探索

### 4.1 新型免疫治疗策略的开发

近年来，许多研究证实免疫治疗在癌症治疗中的应用取得了显著进展，尤其是在NSCLC中。针对*KRAS*突变的肿瘤，研究者们正在探索新型免疫治疗策略，以提高治疗的效果。有研究^[64]^表明，*KRAS*突变型NSCLC患者由于具有适应性免疫抵抗的炎性肿瘤免疫微环境（tumor immune microenvironment, TIME），对ICIs具有更优越的反应。新兴的治疗策略中，结合小分子*KRAS*抑制剂与免疫治疗的方法引起了广泛关注，研究者们期望通过多种机制来增强抗肿瘤免疫反应，优化患者的临床转归。例如，索托拉西布和阿达格拉西布等*KRAS* G12C抑制剂的临床试验显示出与免疫治疗结合的潜力，可能改善患者的生存率和生活质量^[65,66]^。目前除了靶向G12C突变的抑制剂（如索托拉西布和阿达格拉西布）外，*KRAS* G12D抑制剂（如MRTX1133等）也正在开发中，希望为*KRAS*致癌突变的NSCLC群体呈现创新性免疫治疗策略。除了ICIs的使用以外，新辅助免疫治疗同样备受关注，多种免疫通路联合治疗的方案，在疗效上或优于ICIs单药治疗，但也可能出现药物副作用^[67]^，因此还需进一步开发和研究。

### 4.2 *KRAS*突变与其他免疫治疗靶点的联合研究

肺癌组织基因检测结果^[68]^显示*KRAS*变异率较为突出，除*KRAS*外，丝氨酸/苏氨酸蛋白激酶11（serine/threonine protein kinase 11, *STK11*）、Kelch样环氧氯丙烷相关蛋白1（Kelch-like ECH-associated protein 1, *KEAP1*）等突变也经常发生，不同的基因突变类型不仅影响肿瘤的生物学特性，还可能在一定程度上影响NSCLC患者对免疫治疗的反应，从而影响肿瘤患者的预后。现有研究^[69]^提示，具有*KRAS*激活突变与TP53功能缺失突变的病例，有可能对免疫治疗产生更优的应答表现，而STK11和KEAP1共突变的患者则可能需要更为积极的治疗策略。而与STK11和*KRAS*共突变患者则可能表现出较差的免疫反应，提示在选择免疫治疗时需考虑共突变的影响^[70,71]^。与*KRAS*单突变的患者相比，STK11和*KRAS*共突变患者在基因表达和免疫细胞浸润方面表现出不同的免疫特征，获得更差的免疫治疗结果^[72]^。因此，针对*KRAS*突变的联合研究，如与其他免疫靶点（如STK11、TP53等）间相互作用的研究非常值得探索，这不仅能助力我们洞悉肿瘤的复杂特性，更有望为研发更具成效的联合治疗方案提供宝贵思路，进而提高患者的整体生存率。

### 4.3 个性化治疗方案的探索与实施

随着对*KRAS*突变及其在肿瘤进展中的作用深入探索发现，*KRAS*突变与特定的人口学和临床病理特征存在一定的关联，中国人群中 NSCLC患者的*KRAS*突变发生率与东亚国家和美国人群相似，表明NSCLC患者*KRAS*突变的相关性可能存在种族依赖性^[73]^，针对这些特点，个性化治疗方案的探索将成为未来研究的重要方向，患者选择和生物标志物的识别是实现个体化治疗的关键。个体化治疗不仅依赖于肿瘤的基因组特征，还需综合考虑患者的TIME和其他临床特征，如年龄、身体基础状况、过往治疗史以及合并症等，将显著影响临床干预策略的制定和患者生存结局^[41]^。*KRAS*突变的存在往往与患者较差的生存预后相关^[74]^，提示我们在诊疗过程中必须开展*KRAS*基因分型检测，使临床医师能够根据患者特异性的分子特征选择最优治疗手段。*KRAS*多样性突变在免疫治疗反应上存在显著差异，这提示我们在制定治疗方案时需考虑这些因素^[41]^。研发更为精准、高效的生物标志物检测技术迫在眉睫，借助此类先进检测方法，能够在免疫治疗开展前，依据患者独特的生物学特征进行精细化筛选探索如何根据患者*KRAS*基因的突变情况和免疫状态来优化治疗策略，结合大数据和人工智能的分析方法，或许能够为个性化治疗方案的制定提供新的思路和工具，从而提高治疗的有效性和安全性，以实现真正的个性化医疗，从而显著提升免疫治疗的成功率，让更多患者获得更好的预后。

## 5 小结与展望

不可否认的是，*KRAS*突变型NSCLC的免疫治疗领域正经历着快速而深刻的变革。尽管当前的研究进展显著，但这一领域仍面临诸多挑战，如*KRAS*突变与免疫治疗的相互作用机制、*KRAS*突变与其他重要基因（如STK11和KEAP1）共突变对生存的影响等。通过对*KRAS*突变的生物学特性进行深入探讨，我们能够更好地理解其在TIME中的作用，从而为*KRAS*突变型NSCLC患者提供更具针对性的治疗方案。不同类型*KRAS*突变型NSCLC的患者对免疫治疗和放、化疗的疗效和预后各不相同，不同的研究方法也可能会得出相互矛盾的结果，这些差异可能源于研究设计、样本选择及治疗方法等方面的不同。因此，建立统一的研究标准和方法论，并进行多中心的临床试验，将有助于确定*KRAS*突变型NSCLC患者最佳的治疗策略。个体化治疗的理念也逐渐成为治疗*KRAS*突变型NSCLC的重要方向，通过利用生物标志物和基因组学技术，我们可以实现更精细的患者分层，从而优化治疗方案，提高NSCLC患者的疗效和预后。未来的研究应当聚焦于新型免疫治疗策略的开发，特别是针对*KRAS*突变的特异性免疫治疗。同时，结合现有的ICIs和靶向治疗，探索其协同作用的潜力，这将为改善*KRAS*突变的NSCLC患者的生存率和生活质量开辟新的路径。总之，随着我们对*KRAS*突变型NSCLC免疫治疗研究的不断深入，期待能够为患者带来有希望的未来。
